# Novel Biomarkers of Grade I Left Ventricular Diastolic Dysfunction in Type 2 Diabetes Patients with Metabolic-Dysfunction-Associated Steatotic Liver Disease

**DOI:** 10.3390/jcm13195901

**Published:** 2024-10-02

**Authors:** Adina Braha, Bogdan Timar, Viviana Ivan, Monica Micloș Balica, Larisa Dăniluc, Romulus Timar

**Affiliations:** 1 Department of Second Internal Medicine Diabetes, Nutrition, Metabolic Diseases and Systemic Rheumatology, “Victor Babes” University of Medicine and Pharmacy, 300041 Timisoara, Romania; braha.adina@umft.ro (A.B.); timar.romulus@umft.ro (R.T.); 2 Department of Diabetes, Nutrition and Metabolic Diseases Clinic, “Pius Brînzeu” Emergency Clinical County University Hospital, 300723 Timisoara, Romania; 3 Second Department of Internal Medicine-Cardiology Clinic, Victor Babeş University of Medicine and Pharmacy, 300041 Timisoara, Romania; 4 Cardiology Clinic, “Pius Brinzeu” Emergency County Hospital Timisoara, 300723 Timisoara, Romania; 5 Doctoral School, “Victor Babes” University of Medicine and Pharmacy, 300041 Timisoara, Romania

**Keywords:** predictive biomarkers, left ventricular diastolic dysfunction, metabolic-dysfunction-associated liver steatohepatitis, type 2 diabetes mellitus

## Abstract

**Background/Objectives**: Prior research has identified a significant association between heart disease and metabolic-dysfunction-associated steatotic liver disease (MASLD); however, the underlying mechanisms are unclear. This study aimed to identify predictive biomarkers associated with grade I left ventricular diastolic dysfunction (LVDD) in patients with type 2 diabetes mellitus (T2DM). **Methods**: This single-center, cross-sectional study evaluated 73 T2DM patients for grade 1 LVDD and MASLD using 2D echocardiography, tissue analysis, spectral color Doppler, and Fibromax. **Results**: This study analyzed 50 patients (mean age 58.0 ± 11.3 years) with a median diabetes duration of 7 years, abdominal obesity (mean body mass index (BMI) 34.4 ± 5.9 kg/m^2^), and a mean HbA1c of 7.9 ± 1.5%. The prevalence of grade I LVDD, fibrosis, mild steatosis, moderate-to-severe liver steatosis, mild MASLD, and moderate MASLD was 54%, 44%, 14%, 80%, 43%, and 34%, respectively. Regression analysis revealed that grade 1 LVDD was positively associated with age, Fibrotest, α2-macroglobulin, epicardiac adipose tissue (EAT), and negatively associated with lateral s′, E wave, E/e′, E/A, medium E′, and septal e′ (*p* < 0.05 for all). α2-macroglobulin > 1.92 g/L (area under the receiver operating characteristic curve (AUROC) = 0.782, sensitivity 70.4%, specificity 81.2%) and fibrotest score > 0.11 (AUROC 0.766, sensitivity 92.6%, specificity 56.2%) were significant predictors of grade I LVDD. **Conclusions**: Although the underlying mechanisms remain unclear, innovative non-invasive biomarkers, such as α2-macroglobulin or fibrotest, could concurrently indicate liver stiffness and the likelihood of grade I LVDD, an early, asymptomatic HF stage in T2DM patients.

## 1. Introduction

Diabetes mellitus (DM) has emerged as a significant global public health challenge and is the third leading cause of death worldwide. Recent statistical data have revealed a troubling increase in the prevalence of diabetes [1]. Growth acceleration persists after COVID-19, as suboptimal management becomes a potent indicator of more severe consequences of SARS-CoV-2 infections among individuals with type 2 DM (T2DM) [2]. In 2021, Romania documented roughly 1.5 million diagnosed diabetes cases, which is consistent with global trends. Additionally, it was observed that one out of every twelve adults in Romania had diabetes [3]. DM has numerous chronic complications, such as liver steatosis and fibrosis [4], cataracts, glaucoma [5], heart failure (HF) [6], psychiatric disorders [7,8], and a high risk of solid malignancies [9,10,11,12,13]. The biological consequences of this issue are significant because they decrease life and health expectancies due to the negative effects on the quality of life caused by acute and chronic complications. Between 2014 and 2021, the most common complications of DM in Romania were stroke and polyneuropathy, followed by retinopathy. The least common complication is acute myocardial infarction [14]. However, data regarding the prevalence of HF in Romanian patients are lacking.

In recent years, the guidelines of professional associations responsible for diabetes care have recommended personalization of T2DM therapy according to the presence of comorbidities, such as atherosclerotic cardiovascular disease, HF, or chronic kidney disease, in which the indicated molecules are from the class of glucose co-transporter-2 (SGLT2) inhibitors or glucagon-like-peptide-1 (GLP-1) receptor agonists with clinical evidence to reduce the risk of these complications [15]. However, according to new guidelines, only a few patients are currently undergoing treatment. One reason may be the underdiagnosis of comorbidities such as HF.

HF is a medical disorder characterized by diverse underlying etiologies and pathophysiological processes [16]. HF, which affects over 56 million individuals globally, presents a considerable burden on health worldwide, resulting in decreased quality of life, recurrent hospitalizations, elevated healthcare expenditures, and a substantial incidence of premature death [16,17,18]. HF has gained considerable attention in diabetology in recent years primarily because of its critical role of SGLT2 inhibitors in the prevention and treatment of cardiovascular disease (CVD) and HF. It is widely recognized that HF is a common complication associated with diabetes [19]. 

Some different clinical entities and stages in HF make up the complex syndrome. These include HF with reduced ejection fraction (HFrEF), which is defined as an LVEF of 40% or less, HF with mildly reduced ejection fraction (HFmrEF), which is defined as an LVEF ranging from 41% to 49%, and HF with preserved ejection fraction (HFpEF), which is defined as an LVEF of 50% or higher.

Furthermore, distinct phenotypes of ventricular dysfunction in systole (left ventricular systolic dysfunction) and diastole (left ventricular diastolic dysfunction (LVDD) can be identified using echocardiography [20]. Bozkurt et al. proposed a revised classification of the HF continuum based on stages: at risk for HF (stage A, without current or prior symptoms/signs/elevated biomarkers of HF); pre-HF (stage B, without current or prior symptoms or signs of HF, but with one evidence of structural heart disease, abnormal cardiac function, or elevated natriuretic peptide levels); HF (stage C); and advanced HF (Stage D) [21]. Among these, LVDD and HFpEF are the most frequent HF phenotypes in the diabetes population. However, there is no universally accepted estimate of the precise prevalence of the various HF subtypes [22,23].

A comprehensive analysis of the occurrence and likelihood of HF diagnosis and outlook in T2DM patients revealed that the occurrence of LVDD was enhanced by up to 43%, which fluctuated depending on the category. Grade I LVDD is more widespread, accounting for up to 40% of all cases [24]. Since the pathogenesis, therapy, and prognosis of HF differ according to the particular subtype, it is crucial to accurately diagnose the disease and identify those at risk of developing HF. In this respect, prompt diagnosis is paramount [25]. 

A recent systematic review of several studies confirmed that there is a strong association between cardiovascular disease and nonalcoholic fatty liver disease (NAFLD). It is proven that NAFLD affects about a quarter of the population and is an independent risk factor for cardiovascular diseases [26,27]. Specialists in the field have adopted a new definition of NAFLD, namely MASLD, i.e., steatotic liver disease associated with metabolic dysfunction [28]. The implications of this change in cardiac structural and functional abnormalities have not yet been elucidated [29,30,31].

Recent epidemiological data indicate that MASLD is linked to not only accelerated coronary artery disease but also several other detrimental effects on the heart, such as an elevated risk of LVDD and hypertrophy, as well as cardiac valvular calcification and arrhythmias, particularly persistent atrial fibrillation [32,33,34,35,36]. Even when controlling for hypertension, T2DM, and other prevalent cardiometabolic risk factors, a thorough meta-analysis of data from over 11 million middle-aged people showed that MASLD substantially raised the long-term risk of acquiring HF by 1.5-fold [37].

Patients with MASLD had an increased chance of getting LVDD, sharing similar pathophysiological pathways, including insulin resistance, inflammation, and the consequences of visceral obesity. A more integrated approach to treating T2DM patients with these connected health issues and early diagnosis and treatment of MASLD and LVDD reduced cardiovascular risks and improved patient outcomes [38]. 

This research aimed to investigate the relationship between grade 1 LVDD and MASLD in T2DM patients and identify potential predictors of grade 1 LVDD. 

## 2. Materials and Methodology

### 2.1. Study Design and Patients

In this single-center, cross-sectional study, patients were prospectively recruited during their clinical visits between 10 October 2022 and 1 June 2024 from the Diabetes Clinic and Outpatient Diabetes Center of the University Emergency County Hospital Pius Brînzeu Timisoara, Romania. The hospital ethics committee approved this study (332/10 October 2022) as it met the requirements of the Helsinki Declaration (version 2013), respecting the confidentiality of patient data according to the General Data Protection Regulation (GDPR) Compliance. All participants signed a written informed consent form before enrollment in this study. 

Patients were eligible for study enrollment if the following criteria were present: T2DM patients over 18 years of age, without chronic viral or alcoholic hepatitis, with no alcohol misuse, left ventricular ejection fraction (LVEF) > 40%, and an estimated glomerular filtration rate (eGFR) > 30 mL/min. The exclusion criteria were significant cardiovascular valvulopathies, atrial fibrillation, previous known HF or history of ischemic stroke, ongoing or planned pregnancy, lactation, type other than T2DM, underweight with a body mass index (BMI) of <23 kg/m^2^, history of pancreatitis, or refusal to participate in this clinical trial.

This study employed stringent exclusion criteria to create a uniform patient group that accurately represented the MASLD-LVDD relationship. Patients with significant cardiac issues, atrial fibrillation, previously diagnosed HF, chronic viral or alcoholic hepatitis, eGFR < 30 mL/min, and BMI < 23 kg/m^2^ were excluded to minimize confounding factors and focus on individuals with preserved heart function and metabolic dysfunction-related liver steatosis. These exclusions strengthened this study’s findings by ensuring that unrelated health conditions did not influence the MASLD-LVDD association.

### 2.2. Laboratory Tests and Clinical Examinations

Under fasting conditions (minimum 12 h of food rest), the patients underwent routine laboratory tests (glycated hemoglobin (HbA1c), high-density lipoprotein cholesterol (HDLc), low-density lipoprotein cholesterol (LDLc), estimated glomerular filtration rate (eGFR), urinary albumin/creatinine ratio (UACr), total bilirubin (TB), alkaline phosphatase (FAL)) and Fibromax, with the prior signing of a responsibility assumption regarding compliance with the pre-analytical conditions established by the external laboratory and the provision of correct height and weight data. Fibromax is a medical laboratory analysis tool developed by BioPredictive, in which the following five non-invasive tests are combined: FIBROTEST (degree of liver fibrosis), ACTITEST (necroinflammatory activity), STEATOTEST (degree of hepatic steatosis), NASHTEST (presence of nonalcoholic steatohepatitis in patients with dyslipidemia, insulin resistance, diabetes, or obesity), and ASHTEST (degree of liver damage in patients with excessive alcohol consumption). The Fibromax score for evaluating the degree of liver fibrosis was obtained using a calculation algorithm that combined the results of medical laboratory analyses with the patient’s age, gender, weight, and height. Medical laboratory analyses for Fibromax included α2 macroglobulin, apolipoprotein A1, total bilirubin, haptoglobin, gamma-glutamyl transferase (GGT), alanine aminotransferase (ALT), aspartate aminotransferase (AST), glycemia, total cholesterol, and triglycerides. The interpretation of Fibromax results was obtained from medical laboratory analyses entered on the BioPredictive website, where a report was generated with the scores for fibrosis, necroinflammatory activity, hepatic steatosis, nonalcoholic steatohepatitis, and alcoholic steatohepatitis.

We assessed the anthropometric characteristics of the patients by calculating their body mass index (BMI) and visceral adipose tissue (VAT). Abdominal waist circumference was measured using a measuring tape midway between the lower rib and iliac crest, and proximal thigh circumference (proximal thigh C) was measured 15 cm proximal to the superior pole of the patella. The VAT was calculated using the formula VAT (women) = 2.15 × waist circumference − 3.63 × proximal thigh circumference + 1.46 × age + 6.22 × BMI − 92.713, respectively VAT (men)= 6 × waist circumference − 4.41 × proximal thigh circumference + 1.19 × age − 213.65 [39].

### 2.3. Echocardiac Ultrasound Protocol

Two expert cardiologists evaluated cardiac function and epicardiac adipose tissue (EAT) by performing 2D echocardiography and tissue and spectral color Doppler imaging using Esaote S.p.A. MyLabX8 eXP (Via Enrico Melen 77, 16152 Genova, Italy, 2021) [40]. The echocardiographic parameters studied were the left atrium (LA) diameter (mm) in the parasternal incidence, long-axis view, left ventricular ejection fraction (LVEF), EAT measured in the subcostal view, parasternal long axis as the hypoechoic distance between the liver and the right ventricular wall at the end of systole, tricuspid annular plane systolic excursion (TAPSE), global longitudinal strain (GLS), and peak velocities of the early (E) and late (A) phases of mitral valve (MV) inflow during diastole (cm/s). We calculated the E/A ratio; E-wave deceleration time (DTE); MV a′, s′, e′ lateral wall and septum velocities; calculated average E/e′ ratio; ascending aorta (AO); Tricuspid annular plane systolic excursion (TAPSE) assessed the right ventricular function.

The 10-year primary risk of atherosclerotic cardiovascular disease (ASCVD) was estimated using the Pooled Cohort Equation (PCE) calculator. The risk was categorized as low (<5%), borderline (5 to <7.5%), intermediate (≥7.5% to <20%), or high (≥20%) [41,42].

### 2.4. Definitions and Outcomes

We defined grade 1 LVDD according to the diastolic dysfunction grading algorithm from the guide for evaluating left ventricular diastolic function by echocardiography developed by the American Society of Echocardiography and the European Association of Echocardiography [43], using the following criteria: E/A < 0.8, DTE> 200 ms, and average E/e′ ≤ 8. 

MASLD was defined in T2DM patients with liver steatosis and at least one of the five cardiometabolic risk factors [44].

FIBROTEST assesses liver fibrosis and classifies it as no fibrosis (F0), minimal fibrosis (F1), moderate fibrosis (F2), advanced fibrosis (F3), and severe fibrosis (F4). The liver steatosis assessed with STEATOTEST classified as S0, no steatosis (<5% fat overload), S1, mild steatosis (5–33% fat overload), S2S3 clinically significant moderate-to-severe steatosis (34–100% fat overload). Compared to burns, liver inflammation assessed with ACTITEST was classified as A0, A1, A2, or A3, indicating no, minimal, significant, or severe active inflammation, respectively. Alcoholic inflammation was verified using the ASHTEST from H0 to H3. NASHTEST assessed inflammation as an overreaction of the body to an accumulation of fat in the liver, present in metabolic diseases such as diabetes, overweight, and dyslipidemia, and classified it as N0, no NASH, N1, mild NASH, N2, moderate NASH, N3, and severe NASH [45]. According to the new nomenclature, we present the data as MASLD instead of NASH in the present study of T2DM patients who also presented with obesity and dyslipidemia.

### 2.5. Statistical Analysis

The data were statistically analyzed using MedCalc^®^ Statistical Software version 22.016 (MedCalc Software Ltd., Ostend, Belgium; 2023) [46]. To meet the desired statistical constraints for the study outcome, we applied the Shapiro–Wilk test to determine whether the continuous numerical variables were normally distributed. Normally distributed variables were presented as mean and standard deviation, whereas non-parametric variables were presented as median, 25–75 percentiles, or interquartile range (IQR) values. The number of individuals in the class and the percentage of the total subgroup represent the qualitative/nominal variables. To assess disparities in central tendency indicators between groups, we utilized *t*-tests (paired/unpaired) to compare the arithmetic means of parametric variables between two groups and Mann–Whitney U tests to assess medians.

Additionally, we employed chi-squared tests to determine the statistical significance of the differences in proportions between groups. Multivariate and logistic regression analyses were conducted to evaluate the strength of the relationships between the numerical variables. The coefficient of determination (R^2^) was calculated to verify the proportion of variation in the independent variable that generated the variation in the dependent variable. To verify the statistical significance of the analyzed correlations and, consequently, the possibility of generalizing the association in the population, we used the distribution test of t-values, which considers the value of the correlation coefficient and the size of the studied sample. To evaluate the association of LVDD grade I with the factors included in the analysis measured on a continuous scale, we built univariate and multivariate logistic regression models, with potential predictors as the variables mentioned above and outcome as a dichotomous event, grade I LVDD. The variation in the risk of occurrence of the outcome was interpreted by the exponential means of the B (Exp (β)) coefficient of the regression equation, equivalent to the percentage change in the relative value of the risk relative to an increase with a unit of measure in the predictor scale. Using Nagelkerke’s pseudo-coefficient of determination, we demonstrate how the regression model explains the occurrence of a dichotomous event.

To assess the predictive ability of grade I LVDD based on a continuous variable’s value, we conducted “Receiver-Operating Characteristics” (ROC) analyses. The predictive performance was measured using sensitivity and specificity. The optimal threshold value for the predictor was determined using the Youden index. To evaluate the statistical significance of the predictive capability, we compared the area under the ROC (AUROC) curves of the model with those of the nondiscriminant model. For 90% study power, the required sample size for the comparison of the area under the ROC curve with a null hypothesis value was 50 patients, based on an α-level of 0.05, a two-sided β-level of 0.10, with 0.760 AUC expected to be found in this study, a null hypothesis AUC of 0.5, and a ratio of sample sizes in negative/positive groups of ½. 

In our analysis, we determined the 95% confidence interval (CI) and considered a *p*-value of less than 0.05 statistically significant.

## 3. Results

This study included 50 patients, of whom 54% (27/50) were women. Overall, the group had a mean age of 58.0 ± 11.3 years, median diabetes duration of 7 (4;15) years, abdominal obesity (mean BMI 34.4 ± 5.9 kg/m^2^, mean waist 116 ± 11 cm), and a mean HbA1c of 7.9 ± 1.5%. Women tended to have a higher BMI. However, men had more VAT than women (median 55.7 vs. 95.7 cm^2^, *p* = 0.01). Regarding the lipid profile, women presented higher values of apo A1, TG, and LDLc than men (*p* < 0.05); all patients were on statin therapy with atorvastatin or rosuvastatin. However, men had lower median GLS values than women (14.2 vs. 17.4%, *p* = 0.02). Two-dimensional echocardiography and tissue and spectral color Doppler revealed significantly higher values of MV a′ lateral wall and septum velocities and ascending AO in men than in women. The FIBROTEST, ACTITEST, and ASHTEST scores were also significantly higher in men than in women. A comparison of the general characteristics by gender is shown in Table 1.

**Figure 1:**
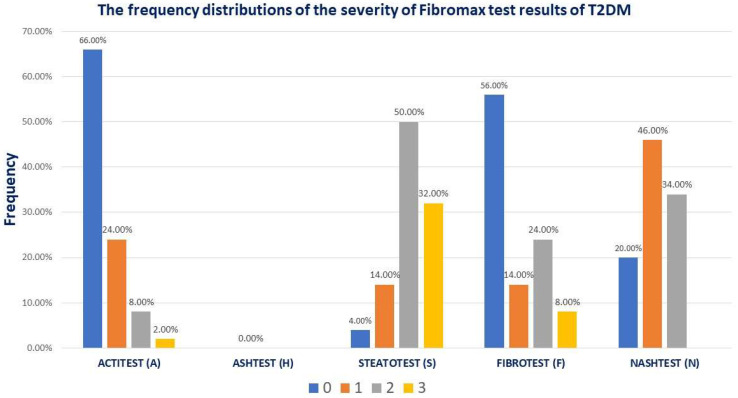
The frequency distribution of the severity of Fibromax test results in studied patients.

The Fibromax scores revealed that 44% had different grades of fibrosis, 14% (7/50) had mild steatosis, 80% (40/50) had moderate-to-severe liver steatosis, 43% (23/50) had mild MASLD, and 34% (17/50) had moderate MASLD (Figure 1).

All patients had normal FEVS and TAPSE and a mean GLS of 16.1 ± 2.5%. A total of 54% (27/50) of the patients had grade I LVDD. Patients with grade I LVDD were older, had higher values in mean VAT (86.2 ± 46.9 cm^2^ vs. 57.7 ± 33.9 cm^2^, *p* = 0.04), mean EAT (4.6 ± 1.5 mm vs. 3.4 ± 1.6 mm, *p* = 0.01), median LA diameter (3.9 cm vs. 3.3 cm, *p* = 0.009), mean ascending AO (2.9 ± 0.3 cm vs. 2.6 ± 0.3 cm, *p* = 0.005), median DTE (219.5 ms vs. 183 ms, *p* < 0.001), median average E/e′ (7.6 ms vs. 6.7 ms, *p* < 0.0001), lower values in median MV e′ septum velocities (7 cm/s vs. 10.5 cm/s, *p* = 0.0001), and MV e′ lateral wall velocities (8 cm/s vs. 12 cm/s, *p* = 0.0001), compared with patients without LVDD. Notably, patients with grade I LVDD had a significantly higher PCE score of 22.8% vs. 10.8%, *p* = 0.02. Regarding Fibromax results, the FIBROTEST score was significantly higher in patients with grade I LVDD than those without, 0.19 vs. 0.11, *p* = 0.003. After applying the chi-square test across the severity of different Fibromax scores, there were no statistical differences in the frequency of grade I LVDD. α2 macroglobulin levels were significantly higher in patients with grade I LVDD (2.2 g/L vs. 1.6 g/L, *p* = 0.002). Table 2 shows the differences among the factors studied in the presence of grade I LVDD.

The regression analysis evaluated the strength of the associations between numerical variables using regression coefficients. For bivariate regressions, we calculated the coefficient of determination (R^2^) to verify the proportion of variation in the independent variable (studied factors) to generate the variation in the dependent variable (grade I LVDD). To verify the statistical significance of the analyzed correlations and, consequently, the possibility of generalizing the association in the population, we used a distribution test of t-values that considered the value of the correlation coefficient and the size of the studied sample. Table 3 presents the results of the regression analysis. There were significant direct associations between the outcome (grade I LVDD) and age, VAT, α2 macroglobulin, EAT, TAPSE, LA, DTE, MV s′ lateral wall velocities, ascending AO, MV A wave velocity, PCE risk, and FIBROTEST score, respectively negative associations with MV e wave velocity, average E/e′, MV e′ septum and lateral wall velocities.

According to the univariate logistic regression model presented in Table 4, age was a significant predictive factor (Exp (β) = 1.25, *p* = 0.001). This result suggests that for every 1-year-old increase, the risk of grade 1 LVDD increased by 25%. For every 1 cm^2^ VAT and every 1 mm EAT increase, the risk of grade 1 LVDD increased by 1% (*p* = 0.04) and 94% (*p* = 0.03), respectively. Similarly, for every 1% increase in PCE risk, the risk of grade 1 LVDD increases by 12%. Increases in MV s” lateral wall velocities, E wave velocities, e′ septum, and lateral wall velocities decrease the likelihood of grade I LVDD. Increases in MV A wave velocities, DTE, average E/e′, α2 macroglobulin, and FIBROTEST scores increased the likelihood of grade I LVDD. 

**Figure 2:**
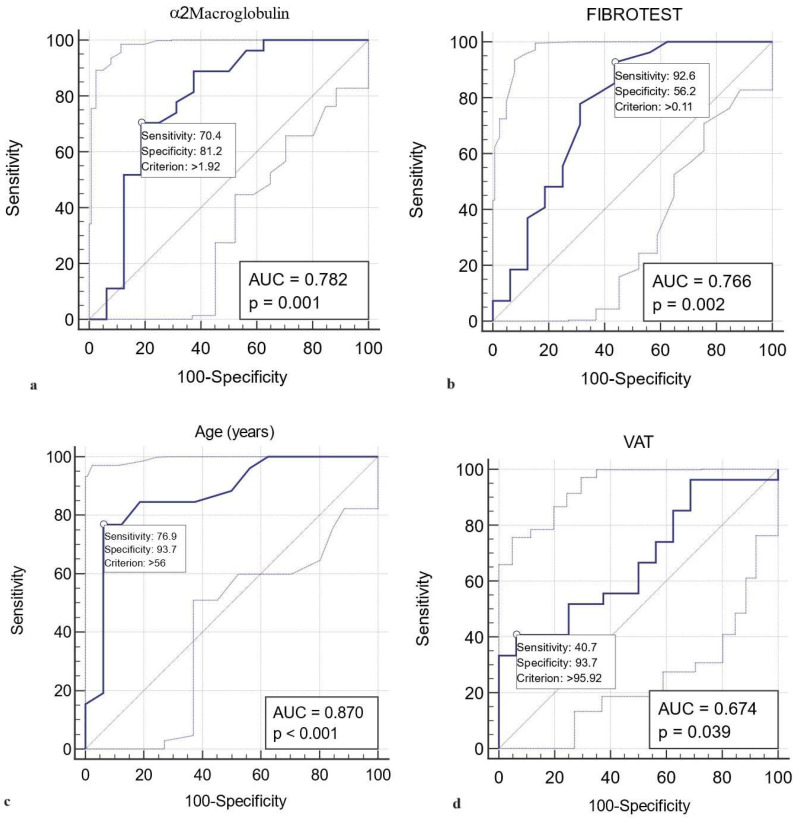
Graphical representation of the ROC curve analysis of the factor thresholds diagnostic ability of grade I LVDD in patients with T2DM and MASLD. **Legend** The subfigures (**a**) (factor α2 macroglobulin > 1.92 g/L), (**b**) (factor Fibrotest > 0.11), (**c**) (factor age > 56 years), and (**d**) (factor VAT > 95.92 cm^2^) shows the thresholds diagnostic ability of grade I LVDD in patients with T2DM and MASLD, for each predictive factor.

A value higher than 1.92 g/L for α2 macroglobulin represented a statistically significant predictive value (AUROC curve = 0.782, sensitivity = 70.4%, specificity = 81.2%, *p* = 0.001) for grade 1 LVDD (Figure 2a). Similarly, FIBROTEST scores above 0.11 predict LVDD grade 1 with a sensitivity of 92.6%, specificity of 56.2%, and AUROC curve = 0.766, *p* = 0.002. The results indicated that FIBROTEST scores above 0.11 correctly report 56.2% of patients without grade 1 LVDD as test-negative (true negatives), but 43.8% of patients without grade 1 LVDD were incorrectly identified as test-positive (false positives) (Figure 2b). Younger T2DM patients than 56 years were unlikely to have grade 1 LVDD (AUROC curve = 0.870, sensitivity = 76.9%, specificity = 93.7%, *p* < 0.001) (Figure 2c). A VAT > 95.9 cm^2^ predicts LVDD grade 1 with a sensitivity of 40.7%, specificity of 93.7%, and AUROC curve = 0.674, *p* = 0.03, indicating that patients with VAT lower than 95.9 cm^2^ will rule out grade 1 LVDD in 93.7% of cases (Figure 2d). 

## 4. Discussion

This study demonstrates the intricate relationship between MASLD and grade I LVDD in individuals with T2DM. This study included 50 people with a mean age of 58 years and a diabetes duration of 7 years. The principal results indicated grade I LVDD in 54% of patients and moderate-to-severe liver steatosis in 80%. The high prevalence of grade I LVDD and MASLD among T2DM patients highlights the need for early diagnosis of cardiac dysfunction in these patients. This study identified α2-macroglobulin and Fibrotest scores as strong predictors of grade I LVDD. These biomarkers may assist in diagnosing asymptomatic pre-HF in people with T2DM.

The FIBROTEST score was significantly higher in the patients with grade I LVDD than those without LVDD. A recent investigation revealed that 73.3% of patients exhibited grade 1 LVDD on cardiac ultrasound evaluation. After a year of dapagliflozin treatment, 24.4% of these patients demonstrated remission of grade 1 LVDD. A significant predictor of favorable therapy response was a decrease in liver fibrosis. Although the study design did not establish causality, it did not delve into the underlying mechanism linking the remission of LVDD and the reduction in liver fibrosis [47].

In our study, patients with grade I LVDD demonstrated a significantly higher PCE score of 22.8% compared to 10.8%. Researchers have been exploring distinct phenogroups within HF to enable more targeted interventions: younger, cardiometabolic phenogroups (predominantly female gender with a high prevalence of cardiometabolic and coronary diseases) and frail phenogroups, which comprise patients with lung disease or atrial fibrillation, respectively, and inflammatory phenogroups comprising patients characterized by systemic inflammation with high rates of diabetes and renal dysfunction [48]. The results of this study delineate a portrait of patients with grade 1 LVDD who concurrently present with MASLD, substantial visceral adipose tissue, and surpass the age of 56 years, accompanied by α2-macroglobulin concentrations higher than 1.92 g/L and liver fibrosis. By univariate logistic regression, we observed that age was a great predictor of grade I LVDD, while other variables showed a weaker association. To test the power of prediction of ɑ2 macroglobulin, FIBROTEST, and VAT for grade I LVDD, we included these variables in a multivariate model aiming to adjust the effect of these variables for the effect of age. After adjusting for age, no factors were retained in the model. An explanation for these results could be a lack of power in the multivariate analysis due to the small sample size. To reliably assess the predictive value of ɑ2 macroglobulin, FIBROTEST, and VAT for grade I LVDD, corrected by age, the sample size should comprise 149 participants to achieve at least 20 events per variable (effect size 0.15, alpha level 0.05, power 0.80), given a prevalence of 54% of grade I LVDD, to avoid overfitting and ensure reliable parameter estimates. It is essential to conduct additional research to explore the correlation between these factors and the potential for implementing targeted therapy for α2-macroglobulin to mitigate the likelihood of grade 1 LVDD.

Patients with grade I LVDD were older and had higher EAT thickness and VAT than those without LVDD. Notably, α2 macroglobulin levels were significantly higher in the patients with grade I LVDD. α2-macroglobulin, a significant plasma glycoprotein primarily produced in the liver, is a large protein synthesized in the body [49] and is believed to be linked to inflammatory responses and properties that promote blood clotting, which could lead to a lack of blood flow. Endothelial dysfunction contributes to the progression of plaque atherosclerosis and the development of cardiovascular diseases [50]. α2-macroglobulin is a protein with diverse inhibitory functions, including the suppression of endopeptidases. It has been linked to various physiological processes, such as cell growth, inflammation, and coagulation. A Moli-sani cohort study demonstrated a relationship between α2-macroglobulin and cardiovascular events. Specifically, individuals with high α2-macroglobulin levels were found to have an increased risk of developing coronary heart disease. Interestingly, this association was observed regardless of the subject’s age, gender, or lifestyle [51]. 

Cardiovascular proteomics has made impressive strides in recent years, with substantial progress in identifying novel biomarkers and elucidating the molecular mechanisms underlying cardiovascular diseases [52]. 

Our data indicated a significant correlation between α2-macroglobulin and grade I LVDD, a finding that has not been previously documented. This publication suggests a pathway for linking liver steatosis and grade I LVDD from a multifactorial viewpoint. Also, it highlights that MASLD is common in people with T2DM and is linked to an increased risk of cardiovascular illnesses, particularly grade I LVDD. According to previous investigations, insulin resistance, inflammation, and visceral fat cause hepatic and cardiac dysfunction [38]. Understanding these relationships may improve patient outcomes and avoid clinical HF.

Significant evidence supports SGLT-2 inhibitor’s management benefits in HFpEF and MASLD. Studies demonstrate that these molecules improve diastolic function, HF hospitalization rates, and cardiovascular outcomes via diuresis, weight reduction, and myocardial energetics. Also, SGLT-2 inhibitors may improve insulin sensitivity and reduce hepatic fat in T2DM patients, delaying liver disease development [53]. 

Identification of predictive biomarkers, such as α2-macroglobulin scores and Fibrotest, provide valuable tools for the early detection of grade I LVDD. Integrating these biomarkers into clinical practice may allow physicians to improve risk stratification and facilitate timely intervention to prevent the progression of symptomatic HF. Moreover, this study emphasizes the need for an awareness of the interconnection between liver and cardiac function, encouraging a more comprehensive approach to patient management. Future research should explore the mechanisms by which MASLD and LVDD influence each other, thus targeting therapies that address both conditions simultaneously, aiming to improve health outcomes for this at-risk patient group.

Several aspects limit the present findings. Among these, we particularly recognize this study’s cross-sectional design, which limits the ability to establish causality by capturing data at a single point rather than across dynamics. In the case of the current research, temporal changes in the relationship between MASLD and LVDD grade I are missing. The small sample size of 50 patients may limit the generalization of the results. Increasing the number of participants in our study would enhance the likelihood that alpha 2 macroglobulin and FIBROTEST will show predictive value for grade I LVDD after adjusting for age. Aiming for a larger sample size, ideally around 500 participants, will provide more robust data, reduce variability in estimates, and improve the overall reliability of our findings. Moreover, a gender-stratified analysis would have been of interest. The single-center nature of this study may also introduce selection bias, as patient demographics and clinical characteristics may not be found in other populations. Although exclusion criteria were used to control for confounding variables, the possibility of residual confounding remains due to unmeasured factors that may affect liver and heart function. Emphasizing the importance of conducting larger, multicenter studies to confirm and further investigate these relationships is essential.

## 5. Conclusions

Our study revealed a significant association between MASLD and grade I LVDD in T2DM patients. Given the high occurrence of LVDD in the T2DM population, it is crucial to identify and address cardiac dysfunction early, even when symptoms are not apparent. Non-invasive biomarkers such as α2-macroglobulin and Fibrotest scores show potential for predicting LVDD and assessing liver stiffness, helping physicians categorize patients based on risk. Rising T2DM and MASLD rates highlight the necessity for integrated care addressing cardiovascular and liver health. Future research should focus on longitudinal studies on larger populations to elucidate causal relationships and assess the efficacy of targeted therapies like SGLT-2 inhibitors in mitigating cardiovascular risks associated with MASLD. Improving our understanding of these links can enhance patient outcomes and quality of life for this vulnerable group.

## Figures and Tables

**Table 1:** General characteristics of the studied patients compared by gender.

Variable	Overall	Women (*n* = 27)	Men (*n* = 23)	*p* ^a^
Age ^a^ (years)	58.0 ± 11.3	57.5 ± 11.5	58.6 ± 11.2	0.7
Diabetes duration ^a^ (years)	7.0 (4.2; 15.0)	8 (21.2)	7 (22.8)	0.6
Abdominal waist ^a^ (cm)	116.0 ± 11.0	115 (22.4)	116 (27.1)	0.2
Hip waist ^a^ (cm)	107.5 ± 11.6	106 (25.7)	105 (22.1)	0.3
BMI ^a^ (kg/m^2^)	34.4 ± 5.9	35.2 (25.5)	33.3 (25.4)	0.9
VAT ^a^ (cm^2^)	75.7 ± 45.1	55.7 (20.7)	95.7 (31.1)	0.01
FG ^a^ (mg/dL)	150.7 ± 42.3	143 (22.4)	158 (29.1)	0.1
HbA1c ^a^ (%)	7.9 ± 1.5	7.9 (22.5)	8.1 (25.7)	0.4
Haptoglobin ^a^	1.7 ± 0.5	1.6 (25)	1.6 (25.9)	0.8
α2 Macroglobulin ^a^	2.0 ± 0.6	2 (26.6)	1.9 (24.1)	0.5
Apo A1 ^a^	1.4 (1.3; 1.6)	1.5 (29.7)	1.3 (20.5)	0.02
TG ^a^ (mg/dL)	162.0 (130.2; 252.2)	153 (25)	157 (26)	0.8
TC ^a^ (mg/dL)	177.6 ± 49.8	170 (29.2)	135 (21.1)	0.04
HDLc ^a^ (mg/dL)	43.0 (36.8; 51.7)	46 (28.8)	40.9 (20.2)	0.03
LDLc ^a^ (mg/dL)	100.7 ± 40.9	109 (28.6)	85 (19.1)	0.01
Uric Acid ^a^ (mg/dL)	5.5 ± 1.2	5.5 (23.5)	5.5 (25.7)	0.5
eGFR ^a^ (mL/min)	87.6 ± 20.7	84.1 (16.6)	88.2 (21.1)	0.2
Serum creatinine ^a^ (mg/dL)	0.7 (0.6; 0.9)	0.7 (21.3)	0.9 (30.3)	0.02
UACr ^a^ (mg/g)	10.7 (3.9; 41.6)	11.1 (19.8)	6.1 (16.2)	0.3
ALT ^a^ (u/L)	29.0 (23.0; 39.0)	26 (22.3)	32 (29.2)	0.09
AST ^a^ (u/L)	20.0 (15.0; 24.0)	20 (24.7)	20 (26.3)	0.7
TB ^a^	0.5 (0.4; 0.6)	0.4 (22.4)	0.5 (29)	0.1
FAL ^a^	76.5 (67.0; 100.0)	87 (19.5)	76 (15.2)	0.2
GGT ^a^	31.0 (20.0; 41.0)	30 (25.4)	32 (25.5)	0.9
EAT ^b^ (mm)	4.0 (3.4; 4.7)	4 ± 1.5	4.3 ± 1.7	0.6
TAPSE ^a^ (cm)	2.6 (2.3; 2.9)	2.5 (20)	2.6 (21.1)	0.7
GLS ^a^ (%)	15.9 (14.5; 18.0)	17.4 (11.5)	14.2 (5.4)	0.02
LVEF ^a^ (%)	57.0 (50.6; 60.5)	56 (21.1)	58 (24.2)	0.4
LA ^a^ (cm)	3.7 ± 0.5	3.6 (19.7)	3.8 (25.1)	0.1
MV s′ lateral wall ^a^ (cm/s)	8.5 (7.0; 10.0)	8 (19.7)	9 (23.8)	0.2
MV s′ septum ^a^ (cm/s)	7.0 (6.0; 8.0)	7 (20.9)	7.5 (23.5)	0.4
DTE ^a^ (ms)	205.0 (188.2; 240.7)	204 (21)	207 (21)	1
MV a′ lateral wall ^a^ (cm/s)	12.0 (9.7; 13.2)	10.5 (19.3)	12 (23.2)	0.2
MV a′ septum ^a^ (cm/s)	10.0 (9.0; 11.0)	10 (18.1)	11 (26.5)	0.02
Ascending AO ^b^ (cm)	2.9 (2.6; 3.2)	2.7 ± 0.4	3.0 ± 0.2	0.04
MV A-wave Vmax ^a^ (cm/s)	80 (68.5; 90.0)	82 (25.1)	76.5 (17.6)	0.05
MV E-wave Vmax ^a^ (cm/s)	65.5 (57.5; 77.5)	67 (22.9)	63 (21.9)	0.8
average E/e′ ^a^	7.1 (6.2; 8.0)	7 (20.6)	7.1 (21.5)	0.8
MV e′ septum ^a^ (cm/s)	8.0 (7.0; 10.0)	8 (23.1)	8.5 (20.4)	0.4
MV e′ lateral wall ^a^ (cm/s)	10.0 (8.0; 13.0)	10 (19.7)	10.5 (23.8)	0.2
PCE risk ^a^ (%)	18.2 (8.5; 33.5)	15.2 (13)	27.9 (17.8)	0.1
FIBROTEST score ^a^	0.1 (0.1; 0.3)	0.1 (21.6)	0.2 (30)	0.04
ACTITEST score ^a^	0.1 (0.0; 0.2)	0.1 (21.3)	0.1 (30.3)	0.03
ASHTEST score ^a^	0.0 (0.0; 0.0)	0.01 (29.2)	0.0 (21)	0.03
NASHTEST score ^a^	0.5 (0.5; 0.7)	0.5 (26.4)	0.5 (23.3)	0.4
STEATOTEST score ^b^	0.6 (0.5; 0.7)	0.6 ± 0.1	0.6 ± 0.1	0.9

Abbreviations: BMI, body mass index; VAT, visceral adipose tissue; FG, fasting glycemia; HbA1c, glycated hemoglobin A1c; Apo A1 = Apolipoprotein AI; TG, triglycerides; TC, total cholesterol; HDLc, high-density lipoprotein cholesterol; LDLc, low-density lipoprotein cholesterol; eGFR, estimated glomerular filtration rate; UACr, urinary albumin/creatinine ratio; ALT, alanine aminotransferase; AST, aspartate aminotransferase; TB, total bilirubin; FAL, phosphatase alkaline; GGT, gamma-glutamyl transferase; EAT, epicardiac adipose tissue; TAPSE, tricuspid annular plane systolic excursion; GLS, global longitudinal strain; LVEF, left ventricle ejection fraction; LA = left atrium; MV, mitral valve; DTE, E-wave de; AO, aorta; PCE risk, pooled cohort risk predictor of the 10-year risk for the first ASCVD event. ^a^ Mann–Whitney test; ^b^ *t*-test for gender comparison.

**Table 2:** Comparison of studied factors by grade I LVDD presence in patients with T2DM.

Variable	With LVDD Grade I (n = 27)	Without LVDD Grade I (n = 23)	∆	*p* ^a^
Age ^a^ (years)	63.5 (27.4)	50.5 (11.8)	−13.1	0.0001
Diabetes duration ^a^ (years)	9 (19.8)	7 (15.7)	−3.5	0.2
Abdominal waist ^a^ (cm)	115 (22.9)	112.5 (19.1)	−3	0.3
Hip waist ^a^ (cm)	104 (20.7)	104 (20.1)	−2	0.8
BMI ^a^ (kg/m^2^)	34.2 (22.7)	34.2 (20.7)	−1.5	0.6
VAT ^b^ (cm^2^)	86.2 ± 46.9	57.7 ± 33.9	−28.4	0.04
FG ^b^ (mg/dL)	145.4 ± 36.6	143 ± 36.3	−2.4	0.8
HbA1c ^a^ (%)	7.7 (17.8)	8.8 (24.9)	0.8	0.06
Haptoglobin ^b^ (g/L)	1.8 ± 0.6	1.5 ± 0.3	−0.2	0.08
α2 Macroglobulin ^a^ (g/L)	2.2 (26.5)	1.6 (14.3)	−0.5	0.002
Apo A1 ^a^ (g/L)	1.4 (22.8)	1.4 (20.5)	−0.0	0.5
TG ^a^ (mg/dL)	117 (20.1)	167.5 (25.1)	8.3	0.2
TC ^b^ (mg/dL)	158.2 ± 47.4	168.6 ± 46.1	10.4	0.4
HDLc ^a^ (mg/dL)	42 (22.3)	42.5 (21.4)	−0.9	0.8
LDLc ^a^ (mg/dL)	91 (18.9)	111.5 (24.2)	15.2	0.1
Uric Acid ^a^ (mg/dL)	5.6 (24)	5.3 (18.4)	−0.3	0.1
eGFR ^b^ (mL/min)	82.7 ± 18.3	95 ± 16.6	12.2	0.06
Serum creatinine ^b^ (mg/dL)	0.8 ± 0.1	0.7 ± 0.1	−0.0	0.6
UACr ^a^ (mg/g)	13 (17)	5.7 (11.1)	−58.3	0.07
ALT ^a^ (u/L)	27 (19.6)	30.5 (25.9)	−3.9	0.1
AST ^b^ (u/L)	22.8 ± 16.7	19.3 ± 4.4	−3.5	0.4
TB ^b^ (µmol/L)	0.6 ± 0.4	0.6 ± 0.3	−0.0	0.9
FAL ^b^	83.9 ± 22.8	81.9 ± 33	−2	0.8
GGT ^a^ (u/L)	29 (21.9)	31 (22.1)	−13.3	0.9
EAT ^b^ (mm)	4.6 ± 1.5	3.4 ± 1.6	−1.2	0.01
TAPSE ^a^ (cm)	2.5 (17.5)	2.8 (23.5)	0.2	0.1
GLS ^a^ (%)	15.5 (8.8)	17 (10.1)	0.7	0.6
LVEF ^a^ (%)	56 (20.6)	56.5 (24.3)	2.0	0.3
LA ^a^ (cm)	3.9 (24.9)	3.3 (14.9)	−0.4	0.009
MV s′ lateral wall ^a^ (cm/s)	8 (16.6)	10 (27.8)	2.2	0.002
MV s′ septum ^a^ (cm/s)	7 (20.4)	7 (23.2)	0.3	0.4
DTE ^a^ (ms)	219.5 (27.6)	183 (9.4)	−56.9	<0.0001
MV a′ lateral wall ^a^ (cm/s)	12 (20.5)	12 (21.6)	0.4	0.7
MV a′ septum ^a^ (cm/s)	10.5 (22.5)	9.5 (19.7)	−0.3	0.4
Ascending AO ^b^ (cm)	2.9 ± 0.3	2.6 ± 0.3	−0.3	0.005
MV A-wave Vmax ^a^ (cm/s)	86 (28)	66 (11.8)	−20.1	<0.0001
MV E-wave Vmax ^a^ (cm/s)	60 (18)	71.5 (28.7)	11.8	<0.0001
average E/e′ ^a^	7.6 (24.4)	6.7 (15.6)	−1.4	<0.0001
MV e′ septum ^a^ (cm/s)	7 (15.6)	10.5 (30.9)	3.5	0.0001
MV e′ lateral wall ^a^ (cm/s)	8 (15.1)	12 (30.1)	3.7	0.0001
PCE risk ^a^ (%)	22.8 (15.1)	10.8 (7.5)	−13.7	0.02
FIBROTEST score ^a^	0.19 (26.2)	0.11 (14.8)	−0.1	0.003
ACTITEST score ^a^	0.1 (21)	0.1 (23.5)	−0.01	0.5
ASHTEST score ^a^	0.01 (24.1)	0 (18.4)	−0.005	0.1
NASHTEST score ^a^	0.5 (22)	0.5 (20.5)	−0.01	0.6
STEATOTEST score ^b^	1.9 ± 0.8	2.1 ± 0.6	0.2	0.3

Abbreviations: ∆, mean difference between group variables; BMI = body mass index; VAT, visceral adipose tissue; FG, fasting glycemia; HbA1c, glycated hemoglobin A1c; Apo A1 = Apolipoprotein AI; TG, triglycerides; TC, total cholesterol; HDLc, high-density lipoprotein cholesterol; LDLc, low-density lipoprotein cholesterol; eGFR, estimated glomerular filtration rate; UACr, urinary albumin/creatinine ratio; ALT, alanine aminotransferase; AST, aspartate aminotransferase; TB, total bilirubin; FAL, phosphatase alkaline; GGT, gamma-glutamyl transferase; EAT, epicardiac adipose tissue; TAPSE, tricuspid annular plane systolic excursion; GLS, global longitudinal strain; LVEF, left ventricle ejection fraction; LA = left atrium; MV, mitral valve; DTE, E-wave deceleration time; AO, aorta; PCE risk, pooled cohort risk predictor of the 10-year risk of the first ASCVD event. ^a^ Mann–Whitney test; ^b^ *t*-test.

**Table 3:** Regression analysis of grade I LVDD and studied factors in patients with T2DM.

Parameter	R^2^	Coefficient	Standard Error	95% CI	t	*p*
Age (years)	0.39	0.03	0.00	0.01–0.04	5.13	<0.0001
Diabetes duration (years)	0.06	0.01	0.01	−0.00–0.04	1.58	0.12
Abdominal waist (cm)	0.02	0.00	0.00	−0.00–0.02	0.91	0.36
Hip waist (cm)	0.00	0.00	0.00	−0.00–0.01	0.51	0.60
BMI (kg/m^2^)	0.01	0.00	0.01	−0.01–0.03	0.75	0.45
VAT (cm^2^)	0.09	0.00	0.00	0.00–0.00	2.11	0.04
TyG index	0.00	−0.16	0.26	−0.69–0.36	−0.62	0.53
FG (mg/dL)	0.01	−0.00	0.00	−0.00–0.00	−0.81	0.42
HbA1c (%)	0.05	−0.06	0.04	−0.16–0.02	−1.50	0.13
Haptoglobin (g/L)	0.06	0.23	0.13	−0.03–0.51	1.75	0.08
α2 Macroglobulin (g/L)	0.16	0.33	0.11	0.09–0.56	2.86	0.006
Apo A1 (g/L)	0.00	0.06	0.27	−0.48–0.62	0.24	0.80
TG (mg/dL)	0.02	0.00	0.00	−0.00–0.00	1.09	0.28
TC (mg/dL)	0.00	−0.00	0.00	−0.00–0.00	−0.54	0.59
HDLc (mg/dL)	0.00	0.00	0.00	−0.01–0.01	0.24	0.80
LDLc (mg/dL)	0.03	−0.00	0.00	−0.00–0.00	−1.24	0.22
Uric Acid (mg/dL)	0.02	0.06	0.06	−0.06–0.18	0.99	0.32
eGFR (mL/min)	0.11	−0.00	0.00	−0.01–0.00	−1.90	0.06
Serum creatinine (mg/dL)	0.00	0.20	0.41	−0.63–1.04	0.50	0.61
UACr (mg/g)	0.05	0.00	0.00	−0.00–0.00	1.23	0.22
ALT (u/L)	0.00	−0.00	0.00	−0.01–0.00	−0.39	0.69
AST (u/L)	0.01	0.00	0.00	−0.00–0.01	0.81	0.41
TB (µmol/L)	0.00	0.02	0.19	−0.37–0.41	0.11	0.90
FAL	0.00	0.00	0.00	−0.00–0.00	0.19	0.84
GGT (u/L)	0.02	0.00	0.00	−0.00–0.00	0.95	0.34
EAT (mm)	0.13	0.10	0.04	0.02–0.19	2.53	0.01
TAPSE (cm)	0.16	0.37	0.13	0.10–0.64	2.70	0.008
GLS (%)	0.02	−0.03	0.05	−0.13–0.07	−0.60	0.55
LVEF (%)	0.02	−0.01	0.01	−0.03–0.01	−1.04	0.30
LA (cm)	0.16	0.37	0.13	0.10–0.64	2.79	0.008
MV s′ lateral wall (cm/s)	0.24	−0.10	0.02	−0.16–0.04	−3.52	0.001
MV s′ septum (cm/s)	0.01	−0.03	0.04	−0.12–0.06	−0.66	0.50
MV a′ lateral wall (cm/s)	0.00	−0.01	0.02	−0.07–0.04	−0.51	0.61
MV a′ septum (cm/s)	0.00	0.01	0.03	−0.04–0.07	0.49	0.62
Ascending AO (cm)	0.17	0.50	0.17	0.15–0.85	2.94	0.005
PCE risk (%)	0.18	0.01	0.00	0.00–0.02	2.26	0.03
FIBROTEST score	0.10	0.94	0.42	0.09–1.80	2.24	0.03
ACTITEST score	0.00	0.21	0.66	−1.12–1.55	0.32	0.74
ASHTEST score	0.07	12.25	6.86	−1.61–26.13	1.78	0.08
NASHTEST score	0.00	0.10	0.44	−0.78–0.99	0.22	0.82
STEATOTEST score	0.01	−0.40	0.49	−1.41–0.60	−0.80	0.42

Abbreviations: LVDD, left ventricular diastolic dysfunction; R^2^, coefficient of determination; BMI, body mass index; VAT, visceral adipose tissue; FG, fasting glycemia; HbA1c, glycated hemoglobin A1c; Apo A1, Apolipoprotein AI; TG, triglycerides; TC, total cholesterol; HDLc, high-density lipoprotein cholesterol; LDLc, low-density lipoprotein cholesterol; eGFR, estimated glomerular filtration rate; UACr, urinary albumin/creatinine ratio; ALT, alanine aminotransferase; AST, aspartate aminotransferase; TB, total bilirubin; FAL, phosphatase alkaline; GGT, gamma-glutamyl transferase; EAT, epicardiac adipose tissue; TAPSE, tricuspid annular plane systolic excursion; GLS, global longitudinal strain; LVEF, left ventricle ejection fraction; LA, left atrium; MV, mitral valve; AO, aorta; PCE risk, pooled cohort risk predictor of the 10-year risk for atherosclerotic cardiovascular disease (ASCVD).

**Table 4:** Logistic regression analysis of risk factors of grade I LVDD.

Variable	Exp (β)	Nagelkerke R^2^	Standard Error	Wald	*p*
Age (years)	1.25	0.54	3.77	10.39	0.001
VAT (cm^2^)	1.01	0.13	0.00	3.85	0.04
α2 Macroglobulin (g/L)	6.05	0.23	1.42	4.49	0.03
EAT (mm)	1.94	0.20	0.31	4.50	0.03
TAPSE (cm)	0.89	0.00	0.21	0.25	0.6
LA (cm)	6.43	0.22	0.77	5.82	0.01
MV s′ lateral wall (cm/s)	0.56	0.31	0.21	7.31	0.006
DTE (ms)	1.17	0.75	0.06	6.86	0.008
Ascending AO (cm)	12.01	0.23	0.98	6.42	0.01
MV e′ septum (cm/s)	0.46	0.51	0.25	9.26	0.002
MV e′ lateral wall (cm/s)	0.53	0.49	0.19	10.49	0.001
PCE risk (%)	1.12	0.32	0.06	3.77	0.05
FIBROTEST score	309.2	0.16	2.88	3.94	0.04

LVDD, left ventricular diastolic dysfunction; R^2^,coefficient of determination; VAT, visceral adipose tissue; EAT, epicardiac adipose tissue; TAPSE, tricuspid annular plane systolic excursion; LA = left atrium; MV, mitral valve; AO, aorta; PCE risk, pooled cohort risk predictor of 10-year risk for a first atherosclerotic cardiovascular disease (ASCVD) event.

## Data Availability

Participants in this study did not grant permission for the public release of their data, which is why supporting information is currently unavailable because of the confidential nature of the research.
